# Utilizing AI-Powered Thematic Analysis: Methodology, Implementation, and Lessons Learned

**DOI:** 10.7759/cureus.85338

**Published:** 2025-06-04

**Authors:** Arif A Cevik, Fikri M Abu-Zidan

**Affiliations:** 1 Department of Internal Medicine, Emergency Medicine Section, College of Medicine and Health Sciences, United Arab Emirates University, Al Ain, ARE; 2 Department of Surgery, College of Medicine and Health Sciences, United Arab Emirates University, Al Ain, ARE

**Keywords:** ai-assisted research, artificial intelligence (ai), braun and clarke’s framework, custom-gpt, large language models (llms), qualitative research, thematic analysis

## Abstract

Artificial intelligence (AI) has the potential to transform healthcare, medical education, and research. Large language models (LLMs) have gained attention for their ability to improve qualitative research by automating data analysis, coding, and thematic interpretation. While prior research evaluates LLMs' performance in qualitative studies, clear guidelines on their implementation remain scarce. This manuscript offers detailed methods with instructions and prompts for using LLMs in qualitative analysis. It provides a clear, step-by-step, practical approach. We developed a customized generative pre-trained transformer (Custom-GPT) based on Braun and Clarke’s six-step thematic analysis framework. The performance of the model was evaluated across three datasets, comparing its outputs with manually generated codes and themes. Triangulation was conducted using Google's NotebookLM. Across the three datasets, the model generated consistent thematic structures that aligned closely with manual coding. However, slight variability in responses, lack of AI decision-making explanations, and requiring repeated prompting during the process were the main challenges. Additional human interventions were required between steps to refine outputs and ensure methodological integrity. LLMs offer promising opportunities to enhance qualitative thematic analysis. However, their limitations emphasize the necessity of human oversight throughout the process. This report highlights the importance of integrating AI tools responsibly, emphasizing methodological rigor, and developing clear guidelines for AI-assisted qualitative research. Future research should explore ethical frameworks, domain-specific LLMs, and advanced prompt engineering techniques to optimize AI’s role in qualitative analysis.

## Introduction

Artificial intelligence (AI) has transformed healthcare by enhancing diagnostic accuracy, enabling personalized treatment, and streamlining operations across medical specialties. Although early AI applications in medicine were limited by the need for large labeled datasets, data bias, and lack of transparency, recent advancements in deep learning have overcome many of these challenges [1]. Deep learning techniques now allow AI models to efficiently process complex and raw data with minimal human input, improving scalability and accuracy [1]. These innovations have broadened AI’s utility from disease detection to key treatments [2]. As AI continues to refine medical diagnostics and treatment, its impact extends beyond clinical applications into medical education, where it is reshaping teaching methodologies and research approaches [3,4].

The recent guidelines by Tolsgaard et al. provided a foundational overview of the use of AI in this area [4]. The guidelines address the growing incorporation of AI in medical education research, offering practical guidance for medical educators on how to conduct and interpret AI-related studies. They define key terminology and identify relevant issues and data suitable for AI applications. The authors highlight AI's potential to automate tasks, enhance efficiency, and provide new analytical tools. However, they also caution against setting unrealistic expectations and emphasize the importance of interdisciplinary collaboration, explainability, and ethical considerations [4]. Furthermore, the guide underscores the necessity of integrating learning sciences and educational theory into the technical development of AI systems, warning against over-reliance on AI technology. While the use of AI in medical education research is on the rise, there is still much that is unknown about this technology and its methodology. A recent guideline addresses the current gap in thorough descriptions of AI systems, including their design, function, and implementation in medical education research [5]. Qualitative analysis of medical education research is crucial for understanding participants' opinions and the reasons behind their behaviors. Recent reports highlight that AI, especially large language models (LLMs), can significantly assist qualitative analysis through their capabilities in data analysis, coding, and thematic analysis [6-8]. The current literature only offers evaluations of performance and general recommendations for using LLMs in qualitative analysis, and many do not thoroughly discuss clear steps for implementation.

This manuscript provides a detailed methodology for employing LLMs in qualitative analysis in medical education research, offering a structured, step-by-step approach to enhance reliability and reproducibility. By documenting our methodology and lessons learned, this manuscript serves as a practical guide for educators and researchers seeking to integrate LLMs into their qualitative analysis workflows.

## Technical report

Methods

This methodology was developed to support the qualitative analysis of learner data collected from a series of online courses delivered under the International Emergency Medicine (iEM) Education Project.

Setting

The iEM Education Project is a non-profit global initiative that provides free online educational resources for medical students, interns, and educators. Launched in 2018 with the support of the United Arab Emirates (UAE) University College of Medicine and Health Sciences and the endorsement of the International Federation for Emergency Medicine, this project aims to enhance emergency medicine education of medical students and interns worldwide (iem-student.org).

During the COVID-19 pandemic, the project took on a social responsibility initiative by launching a course platform to assist trainees affected by the disruption of educational activities due to the pandemic (iem-course.org). From May 2020 to December 2023, the platform offered five courses: the iEM/Lecturio Emergency Medicine Core Content Course, Extended Focused Assessment with Sonography for Trauma, Rapid Ultrasound in Shock and Hypotension, iEM/Lecturio - COVID-19 Clinical Readiness Course, and Fundamentals of Research in Medicine. The course platform is hosted on a WordPress-based website and utilizes the LearnDash learning management system.

Data Collection

To assess the usage and demand for the courses, a mixed-method research strategy was employed for all courses. The course evaluation studies were exempt from ethical review according to UAE University guidelines, as they did not involve sensitive or identifiable data (ERS_2020_6130). Participants were informed that their involvement was voluntary, and reminders were sent out prior to the course and before each survey.

Data collected from participants involved several steps. Some of this information is gathered as a standard data set through WordPress and LearnDash, in addition to the quantitative and qualitative data collected from course evaluations. Access to these data is restricted to the admin of the website, who is the project director and primary author of this report.

The typical process of data collection in courses begins with visiting the website. Participants select a course based on their preferences and then enroll. During this process, WordPress and LearnDash generate a unique nine-digit user identification number for each participant, which allows for monitoring of all activities associated with the course.

After an orientation session, participants have the option to complete an entry survey that focuses on the course content and objectives. This survey collects only quantitative data. If applicable, a formative knowledge quiz follows.

The next phase involves course delivery, which includes videos, texts, and mini quizzes. Upon completing the course delivery process, participants take another formative knowledge quiz. At this stage, an optional exit survey is offered. This survey collects both quantitative and qualitative data. The qualitative data mainly comes from questions regarding course delivery, content, areas for improvement, and additional comments.

**Figure 1:**
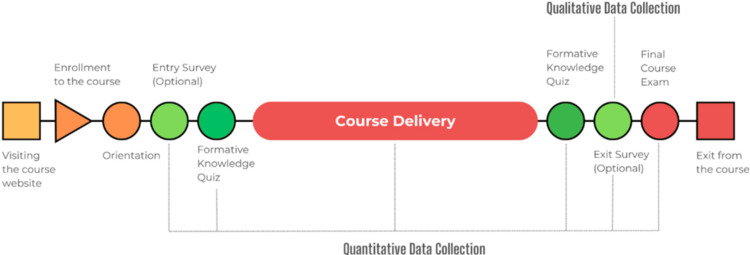
Illustration of the overall sequence of steps in the courses and the locations for data collection. The diagram illustrates the typical flow of a course delivery framework, highlighting key learner interactions from initial engagement to course completion. It starts with visiting the course website and proceeds through enrollment, orientation, and pre-course assessments, followed by the main course delivery phase. Post-course assessments and feedback collection conclude the process. The framework includes both quantitative data collection points (e.g., formative quizzes and final exam) and qualitative data collection opportunities (exit survey). Illustrated by AA Cevik

Finally, participants take the final course exam and complete the course, achieving a predefined cutoff score to pass. Figure 1 illustrates the overall sequence of steps in the courses and the locations for data collection.

Preparation of Data and Utilizing LLMs in Qualitative Analysis

**Figure 2:**
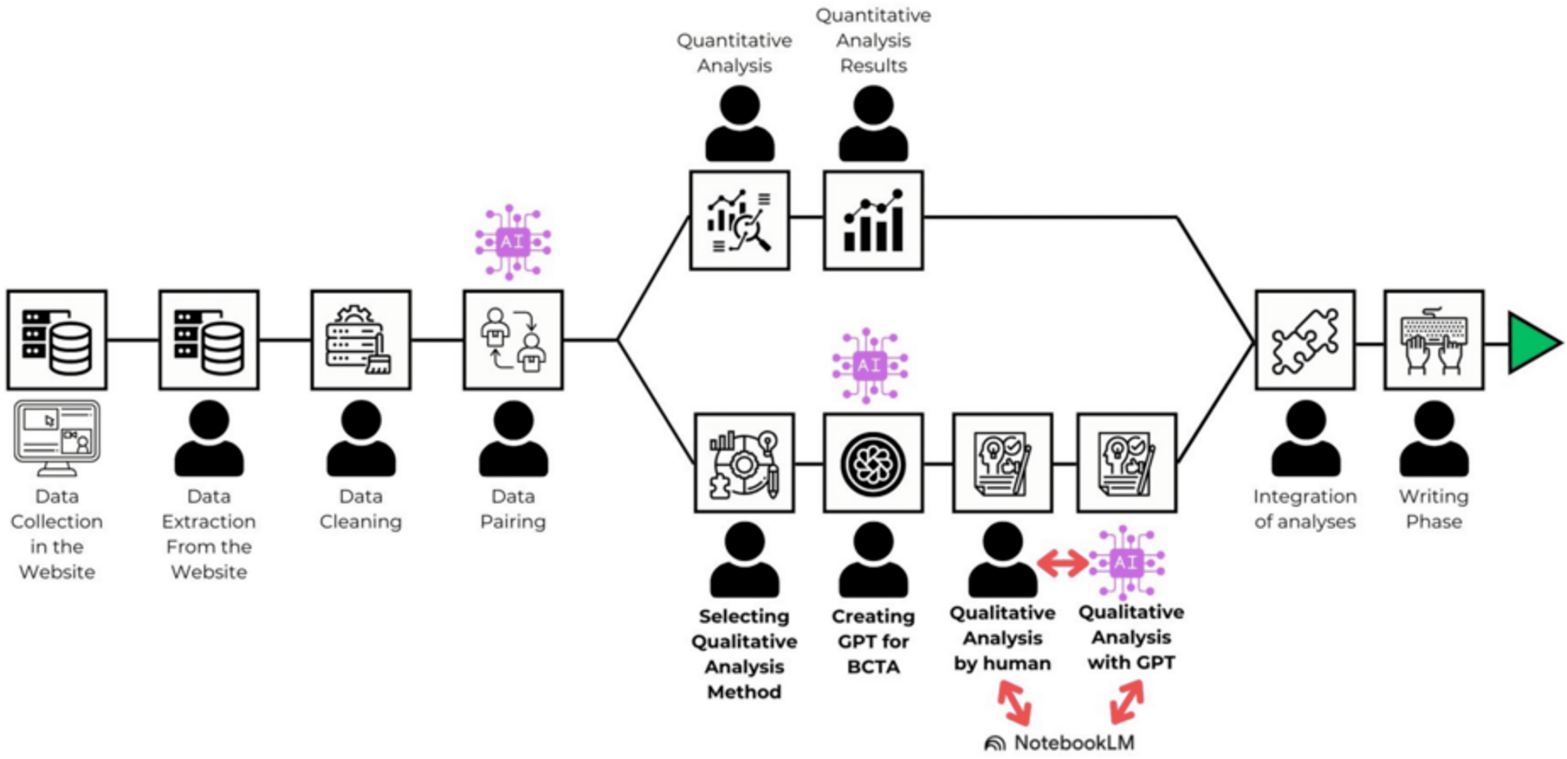
Illustration of steps following data collection. Human graphic represents human involvement. AI: Artificial intelligence, GPT: Generative pre-trained transformer, BCTA: Braun & Clarke’s Thematic Analysis Framework Illustrated by AA Cevik

Before conducting qualitative data analysis using the LLM tools, several steps need to be taken (Figure 2).

Data Extraction and Cleaning

As previously mentioned, data are regularly collected by WordPress and LearnDash through course activity. These data are extracted from the platform in comma-separated values format. The data file is then cleaned to remove any potential identifying information, with the unique nine-digit user identification number being the only item retained that directly relates to the anonymous data.

Selecting LLMs

LLMs are generative AI systems that use natural language processing and are trained on vast amounts of textual data. These models employ advanced machine-learning neural networks that can learn from this information and generate responses [9]. When given prompts, LLMs can engage in conversations and perform various tasks. However, it is important to note that LLMs do not possess human-level cognition. They lack a human-like understanding of the world [10]. We chose ChatGPT from OpenAI and NotebookLM from Google for our qualitative analysis processes. There are several open-access LLMs currently available for public use. Among these, ChatGPT stands out as a widely used tool that is being explored and applied in various aspects of qualitative analysis [9]. This includes inductive and deductive coding, thematic analysis, and even support for other stages of the research processes [8,11]. Selecting a well-researched LLM was the best choice for our analysis. However, very few articles in the literature specifically investigate NotebookLM [12]. NotebookLM is a retrieval-augmented generation tool that helps LLMs to reference reliable information, which is mainly uploaded as an attachment [12]. NotebookLM acts as a virtual research assistant that analyzes uploaded documents and provides insights based solely on those sources. It provides highly accurate reference locations based on reliable external information [12]. NotebookLM specializes in analyzing sources uploaded by researchers, creating a tailored AI assistant that becomes an expert in those specific materials [12]. This source-focused approach ensures that the tool's outputs are directly linked to the researcher's primary materials instead of relying on general knowledge [12]. This makes it especially useful for triangulation processes that require systematic comparisons across multiple data sources.

Data Pairing Using ChatGPT

The next stage involves pairing each participant's pre-course (quantitative) and post-course (both quantitative and qualitative) data. Many researchers typically prefer to combine multiple data files into one by using a common column in Excel with vertical lookup and extended lookup functions, which can be quite efficient. However, instead of Excel, we utilized one of the pioneers of open-access LLMs, ChatGPT. ChatGPT's data analysis capabilities to pair the data based on the nine-digit user ID. When combining two Excel files based on a common column, ChatGPT uses the Code Interpreter (also known as Advanced Data Analysis) function behind the scenes, enabling tasks such as file management. The process involves several key steps to ensure accurate data integration. First, both files are loaded into a structured format, and their column names and data types are examined to confirm compatibility. The next step is identifying the shared column, ensuring that it exists in both datasets and has a consistent data type. Depending on the objective, different techniques can be applied. An inner join merging technique retains only the rows where the common column appears in both files. After merging, it is essential to address duplicates and missing values to maintain data integrity. Finally, the refined dataset can be saved in a new file for further analysis. This systematic approach ensures that data from multiple sources is effectively integrated while preserving accuracy and completeness in the ChatGPT process. We used the inner join option to compare pre- and post-course differences in the same participant. This approach was effective in combining the pre- and post-course data files. The paired data file was then downloaded from ChatGPT in the XLSX format. To ensure the accuracy of the paired data, one of the researchers reviewed the results.

Selecting a Qualitative Analysis Method

Inductive thematic analysis, which is a data-driven method, is advantageous for surveys since it enables themes to arise straight from the data, offering a comprehensive understanding of the overall information [13]. This approach is especially beneficial when examining the experiences, viewpoints, and comprehension of participants regarding a specific topic [7]. ChatGPT is capable of carrying out thematic analysis and executing inductive thematic analysis following Braun and Clarke’s framework [9,14]. Braun and Clarke's six-step process is a widely recognized and commonly used method in qualitative research [7,14,15]. Their approach consists of six progressive phases: familiarizing oneself with the data, generating initial codes, searching for themes, reviewing the themes, defining and naming the themes, and producing a report [14,15]. This method is appreciated for its clear and distinct steps, which make the analytical process easily verifiable [7,14]. Our survey data primarily contain experiences and viewpoints about the courses. As previously tested in ChatGPT [9], we decided to use Braun and Clarke's universally recognized six-step method for our data analysis while utilizing ChatGPT.

Developing Customized Generative Pre-trained Transformers (GPT) for Braun and Clarke's Thematic Analysis Framework

After deciding on the thematic analysis framework, we aimed to create a customized GPT (Custom-GPT) based on that framework. Regular ChatGPT is a versatile AI model designed to handle a wide range of general-purpose tasks without requiring any customization. It is fast and user-friendly and allows for generating responses on the go without prior setup. However, while it offers flexibility, it may need manual input for specific tasks. Maintaining consistency across responses can be challenging. In contrast, Custom-GPTs are tailored versions of the base model, fine-tuned with additional instructions or integrated with external data sources to meet specialized needs. This customization enables ChatGPT to produce highly consistent and specialized outputs, such as structured question formats or domain-specific content, without requiring repeated manual prompts. Moreover, Custom-GPTs can automate workflows and ensure uniformity in generated content [16]. However, creating a Custom-GPT requires an initial setup and refinement process, which can be time-consuming, and it may be less interactive if dynamic adjustments are necessary. While Custom-GPTs perform well in specialized domains, their effectiveness ultimately depends on the quality of their fine-tuning and the specificity of the training data used.

To be eligible to use the Custom-GPT option, we registered as a paid user of ChatGPT for the "Plus" option, which costs 20 USD per month. Before creating a specific GPT, we tested several publicly available Custom-GPTs for qualitative analysis to understand their outputs and potential. These Custom-GPTs can be found on ChatGPT's GPTs page (chatgpt.com/gpts). As of today, there are hundreds of qualitative research assistant GPTs available on this platform for users. Since we could not evaluate the instructions and suggested prompts used in these GPTs, we needed to create our own Custom-GPT and prompts related to the thematic analysis framework.

We have engaged with ChatGPT-4o to grasp its capabilities regarding our intended project and the framework we planned to use. ChatGPT-4o offered detailed insights into Braun & Clarke's framework for thematic analysis. This understanding aligned closely with the information presented in the literature [14].

The next action was to request an outline from ChatGPT-4o for creating a Custom-GPT, utilizing the six-step approach of the framework to evaluate qualitative data. The outline for Custom-GPT Development was as follows: Step 1: Define the purpose of your Custom-GPT; Step 2: Set up your Custom-GPT; Step 3: Configure Braun & Clarke’s six-step process; Step 4: Enable uploads for qualitative data; and Step 5: Test and adjust the output.

Step 1 - Define the Purpose of Your Custom-GPT

We have defined the purpose of Custom-GPT as follows: “This customized GPT is designed to assist researchers in following Braun & Clarke’s six-step process for thematic analysis. It provides structured support for identifying, coding, and organizing themes from qualitative texts, such as interview transcripts and survey responses. The tool promotes rigorous and consistent analysis by encouraging reflexivity and maintaining methodological transparency at each stage. By offering structured outputs, it helps users navigate the thematic analysis process systematically. This resource is designed for medical educators analyzing trainee feedback, social scientists conducting interviews and focus groups, and anyone involved in qualitative research who seeks a reliable and well-structured approach to thematic analysis."

Step 2 - Set Up Your Custom-GPT

To set up a Custom-GPT, we first opened OpenAI’s Custom-GPT Builder. We started by visiting and navigating to the “Explore GPTs” section. From there, we selected “Create a GPT” to begin building a Custom-GPT tailored to our specific needs. We customized the GPT by providing specific instructions to enhance its functionality for qualitative analysis. We named it "AI-Powered Thematic Analysis" and defined its role as follows: “You are an expert in qualitative research and thematic analysis, with 20 years of experience following Braun and Clarke’s six-step framework. Your role is to assist researchers in coding, categorizing, and synthesizing themes from qualitative data through each step with clear explanations while ensuring that the analysis remains transparent, rigorous, and reflexive.”

Step 3 - Configure Braun & Clarke’s Six-Step Process

We provided a clear, step-by-step overview to ensure a structured approach to thematic analysis using Braun & Clarke’s six-step framework. We follow the framework through each step:

Familiarize with the data: Start by reading and engaging deeply with the qualitative text. Summarize the dataset and identify initial patterns, emotions, and key phrases. Reflect on the data by asking reflexivity questions (e.g., "What assumptions do I bring to this data?").

Generate initial codes: Identify meaningful patterns by extracting key phrases and concepts from the text. Assign descriptive labels, known as open codes, to these patterns. Please explain the reasoning behind your choices for generating these initial codes.

Identify broader themes: Look for relationships between the codes and group related codes into overarching themes. Please explain the reasoning behind your choices for identifying these specific themes.

Review themes for coherence: Validate that the themes are distinct, coherent, consistent, and well-supported by the data.

Define and name themes: Provide clear, concise names for each theme. Write a definition for each theme and refine the descriptions to clearly capture their essence. Please provide a detailed explanation and meaning of the themes. Please explain the reasoning behind your definitions and names for these specific themes.

Generate a structured report:Present your findings, including themes, supporting data, and justifications, to ensure transparency and rigor. In addition to text, please provide a table covering themes, explanations, and meanings of the themes, related codes, and related quotes. Make sure the findings align with the research questions.

We also defined how the Custom-GPT should respond dynamically to enhance user engagement and ensure a rigorous thematic analysis process with additional prompts: (1) "Prompt reflexivity by asking the user relevant questions at each step, ensuring they critically engage with the data and justify their thematic choices." (2) "Request user feedback at each stage to refine the analysis and adjust the process as needed." (3) "Dynamically adjust responses based on user input, making the interaction more adaptive and responsive to specific research needs." (4) "Allow interactive questions from the user at any stage, fostering a two-way dialogue that supports deeper analytical thinking." (5) “You can verify your approach by visiting the file attached in the knowledge section.”

Step 4 - Enable Uploads for Qualitative Data

To enable the Custom-GPT to analyze large datasets efficiently, we activated key functionalities, including PDF/text uploads, code interpreter, and data analysis. These features allow the GPT to (1) extract and process qualitative themes automatically from large bodies of text, such as interview transcripts, survey responses, and reports; 2) analyze uploaded PDF and text files, ensuring that users can work with extensive datasets without manual entry; and 3) utilize the code interpreter and data analysis tools to identify patterns, categorize responses, and structure findings efficiently. Our goal in integrating these capabilities is that GPT can handle complex qualitative data, making thematic analysis more scalable, structured, and automated without compromising methodological rigor.

Step 5 - Test and Adjust the Output

We tested the Custom-GPT by running sample qualitative datasets through it and refining its prompt structure to ensure it accurately followed Braun & Clarke’s six-step framework. This approach has been supported in the literature [9,17]. During this process, we focused on three key areas: (1) prompt atructure - adjusting how the GPT guides users through each step, ensuring clarity and adherence to thematic analysis principles; 2) thematic outputs - checking whether the generated themes were coherent, well-defined, and accurately represented the data; and 3) reflexivity prompts - ensuring that the GPT actively asks users to consider potential biases, justify coding decisions, and reflect on their analytical approach.

The manual thematic analysis (Braun & Clarke's framework) was conducted independently and completed before applying the AI-powered thematic analysis. This sequence ensured that human researchers approached and understood the data without influence from the AI-generated outputs.

Test 1

We had the opportunity to test a custom version of ChatGPT for qualitative thematic analysis on the qualitative data from three courses. For our first trial, we decided to start with the course that had the fewest participants: the Research Fundamentals in Medicine course. This course had 272 enrolled participants; however, only 168 started, and 52 completed the entire course process. Out of those 52 students, only 33 provided qualitative feedback. The total word count of this feedback was 762, which is considerably lower compared to the large volumes of data that ChatGPT-4 can process in seconds. Nonetheless, this was a valuable opportunity for us to review all the comments, identify codes and themes, and find relevant supportive data and quotes for each theme. Using these data, we anticipated that Custom-GPT would perform a proper analysis that we could then evaluate. Additionally, to triangulate this analysis, we used NotebookLM by Google to ask specific questions about the data and results of Custom-GPT (Figure 2).

In this exercise, the manual coding produced 21 codes and identified four themes. We conducted three attempts using the same data in Custom-GPT, and each time we received very similar analyses regarding codes and themes. The prompts seemed to work initially well. The thematic outputs satisfied us and accurately represented the data, aligning with our findings. However, Custom-GPT did not provide reflexivity prompts. Custom-GPT generated 25 codes and five themes. We later combined two of the themes to create a final version. Please refer to the results from the manual and the custom GPT, as illustrated in Table 1. Despite the time manually spent on code generation and theme development being relatively short due to the size of the data, we were impressed by how quickly Custom-GPT achieved results comparable to ours.

**Table 1:** The results from the manual and the custom GPT. The table presents codes and themes extracted from qualitative data using manual methods and custom GPT.

Initial Manual Codes (21 Codes)	Custom-GPT Codes (25 Codes)
Clear points. Simple terms. Step-by-step. Easy language. Not confusing. Knows topic. Confident speaker. Good answers. Shares tips. Real examples. Knows what works. Good flow. On schedule. Clear plan. Logical order. Time well used. Real cases. Show how. Practice examples. Use pictures. More videos	Clear explanations. Easy-to-follow. Stepwise approach. Simple language. Logical flow. Instructor expertise. Deep knowledge. Confident delivery. Insightful comments. Practical tips. Structured content. Well-paced. Logical sequence. Time management. Organized delivery. More diagrams. Interactive elements. Engaging visuals. Practical demonstrations. More visual tools. Real-life studies. Clinical research examples. Hands-on learning. Common research oriented. Practical relevance
Initial Manual Themes (4 Themes)	Custom-GPT Themes (5 Themes)
Easy to Understand, Experienced speaker, Well-structured course, More real-life examples and more visuals	Clarity and Simplicity in Teaching, Appreciation for Expert Knowledge, Course Organization and Structure, The Need for Additional Visual and Interactive Materials, Desire for Real-World Application and Clinical Relevance
Final Themes (4 Themes)	
Clarity and Simplicity in Teaching, Appreciation for Expert Knowledge, Course Organization and Structure, The Need for Additional Visual and Interactive Materials	

Test 2

The next test was the Rapid Ultrasound in Shock and Hypotension course, which had 1,008 enrollees. Out of those, 982 started the course, 679 completed it, and only 105 provided qualitative feedback. The total word count was 1,401. In this dataset, we manually evaluated 30% of the participants' qualitative feedback, extracting codes, themes, relevant quotes, and information. The manual coding produced 12 codes and identified four themes. We saved these data to compare it with Custom-GPT results. Additionally, we continued to manually assess the complete qualitative data, manually extracting codes, themes, and relevant quotes and information. Both the 30% sample and the full manual results were compared to those generated by Custom-GPT. In the first attempt, Custom-GPT extracted 11 codes and five themes. In the second attempt, it provided eight codes and six themes. The content of these codes and themes was similar and could easily be combined. The manually extracted codes and themes from the 30% sample of the qualitative data were captured by Custom-GPT. The codes and themes manually generated from the full dataset were also captured by Custom-GPT, with a greater number of codes and themes. By cross-referencing the outcomes from the Custom-GPT with NotebookLM, we obtained sufficient responses and successfully identified the pertinent codes and information within the document. This exercise concluded with the final decision of nine codes and five themes. Please refer to the results from the manual and the custom GPT, as illustrated in Table 2. In this activity, the prompts appeared to be effective up to a certain extent. The thematic outputs met our expectations and appropriately reflected the data, consistent with our findings. Nevertheless, Custom-GPT once again failed to offer reflexivity prompts.

**Table 2:** Test 2 - The results from the manual and the custom GPT. The table presents codes and themes extracted from qualitative data using manual methods and custom GPT.

Initial Manual Codes (12 Codes)	Custom-GPT Codes (11 Codes)
Enjoyed sessions. Suggest to others. Interested in more. Clear explanations. Simple to understand. Stayed focused. Hands-on work. Real examples. Useful comments. Assessments. Quick access. Materials access	Enjoyed course. Would recommend. Wants more topics. Clear message. Easy to follow. Kept attention. Practical tasks. Real-life practice. Helpful feedback. Evaluation/Assessment. Need for easy access
Initial Manual Themes (4 Themes)	Custom-GPT Themes (5 Themes)
Satisfied and Curious to Learn More, Clear and Interesting Teaching, Importance of Learning by Doing, Need for Efficient Testing and Learning Materials	Learner Satisfaction and Future Expansion, Engaging and Clear Content Delivery, Hands-on Learning Experience, Effective Assessment Strategies, Access to Learning Resources
Final Themes (5 Themes)	
Learner Satisfaction and Future Expansion, Engaging and Clear Content Delivery, Hands-on Learning Experience, Effective Assessment Strategies, Access to Learning Resources.	

Test 3

The third test involved the qualitative data from the Extended Focused Assessment with Sonography for Trauma (EFAST) course. In total, 1,758 people enrolled in the course; 1,515 from 111 countries started it; and 1,190 completed all course processes. Out of those 1,190 participants, 220 provided qualitative feedback, resulting in a total of 2,876 words of data.

We performed a manual analysis of these data, focused primarily on a randomly selected 30% of the participants' qualitative feedback. From this subset, we generated codes and themes. We applied our Custom-GPT model to analyze these data. The manual coding produced 16 codes and identified five themes. When we compared our results with those produced by Custom-GPT, we found that all the codes we extracted were captured by the model. However, some codes were represented by synonyms in the Custom-GPT analysis.

In the first attempt, Custom-GPT extracted eight codes and developed five themes from them. In the second attempt, it identified 14 codes, but the themes remained the same. We later combined themes and finalized three themes. Please refer to the results from the manual and the custom GPT, as illustrated in Table 3. When we triangulated the results from Custom-GPT with NotebookLM, we received satisfactory answers and were able to locate the relevant codes and information within the document. In this task, the prompts seemed to be effective. The thematic results aligned with our expectations and accurately represented the data, in line with our observations. However, Custom-GPT once again did not provide users with reflexivity prompts. Similar to the other two datasets, we needed to write additional prompts in the chatbox to improve the results.

**Table 3:** Test 3 - The results from the manual and the custom GPT. The table presents codes and themes extracted from qualitative data using manual methods and custom GPT.

Initial Manual Codes (16 Codes)	Custom-GPT Codes (14 Codes)
Clear voice. Easy language in videos and text. Step-by-step. Well-paced delivery. Engaging images. Active participation. Good flow. Examples included. Better connection. Tech issues. Structured content. User-friendly. Easy to access. Simple layout. Learned a lot. Enjoyed experience	Clear speech. Simple language. Easy to understand. Interactive session. Kept interest. Good visuals. Platform issues. Audio problems. Better layout. Easy login. Smooth navigation. Mobile-friendly. Enjoyed learning. Felt confident
Initial Manual Themes (5 Themes)	Custom-GPT Themes (5 Themes)
Clear Explanations and Speaking Style, Interactive and Well-Presented Sections, Platform Functionality and Course Setup, Ease of Use and Navigation, Enjoyable and Useful Learning	Clarity and Communication Style, Content Delivery and Engagement, Technical and Structural Improvements, User Access and Course Design, Positive Learning Experience
Final Themes (3 Themes)	
Content Delivery and Engagement, Technical and Structural Improvements, Positive Learning Experience	

Triangulation With NotebookLM

The final set of themes and supporting quotes was determined after the triangulation process. Triangulation served as a critical step in refining the initial outputs from both the manual and AI-powered analyses. While the custom-GPT model had already captured the majority of the codes and themes, triangulation allowed us to reconcile differences, enhance clarity, and ensure conceptual coherence. Minor adjustments, such as consolidating overlapping codes or refining theme boundaries, were made during this stage to strengthen the analytical framework.

Depending on the steps of the process, various prompts were used in NotebookLM to triangulate findings from a dataset. Here are some example prompts for NotebookLM: (1) "Based on the provided dataset, do the following themes (list themes) emerge consistently?" (2) "Are there any alternative interpretations?" (3) "Compare the themes given here (list themes) with the core ideas in the dataset. Do they align, or are there discrepancies?" (4) "Analyze the dataset and suggest any potential (codes, themes, or sub-themes) that may not have been explicitly mentioned in this thematic analysis." (5) "Are there any nuances, contradictions, or unexpected findings in the data that do not fit neatly into the identified codes or themes?" (6) "Evaluate the clarity and coherence of these codes/themes: (list codes/themes). Are there overlaps or inconsistencies that need refinement based on this dataset?" (7) "Do these (codes, themes) reflect distinct patterns, or should any of them be merged, split, or refined for better accuracy?" (8) "Compare the themes from my thematic analysis with findings from the dataset. Do similar codes or themes emerge, or are there notable differences?" (9) "Analyze dataset (X) and dataset (Y). Do they share common themes, or do they highlight different perspectives?" (10) "Does this quote represent the (code/theme) in this dataset? Please explain how it relates." (11) "Can the definition ("definition") of this (code/theme) be extracted from this dataset? Please explain how it relates." (12) "If NotebookLM suggests new codes, themes, or sub-themes: Can you provide examples from the dataset that support these codes, themes, sub-themes?" (13) "If it finds contradictions: What possible explanations exist for these contradictions in the data?"

## Discussion

Challenges and solutions

One observation we made was that Custom-GPT did not follow the instructions as precisely as we had written them. As a result, we needed to make additional requests in the chat. For example, we prompted Custom-GPT in the instructions to explain its reasoning behind the decisions made during the process. However, Custom-GPT rarely provided its reasoning initially; we had to repeatedly ask for clarification on its selections from the text. It is mentioned that Custom-GPTs often struggle to follow instructions effectively, especially when using the GPT-4o model. This issue has been widely reported by users, who note that GPT-4o frequently ignores specific directives, leading to inconsistent and unreliable outputs [16]. One theory is that the shift from GPT-4 to GPT-4o has introduced challenges in maintaining consistent behavior. Users have observed that Custom-GPTs, which previously followed instructions accurately, now exhibit unpredictable behavior, suggesting that the new model may handle instructions differently [16]. To address these issues, the following strategies are recommended [16]. First, simplifying instructions is essential to ensure clarity and improve compliance accuracy. Instructions should be clear, concise, and explicitly stated within each prompt to reinforce the desired behavior, even if repetition is necessary. Second, iterative testing should be employed to continuously refine prompts by identifying patterns of misbehavior and adjusting instructions accordingly. Engaging in a back-and-forth dialogue with the model allows for gradual improvements, guiding it toward the desired outcome.

The quality of output generated by ChatGPT is greatly affected by the prompts provided to it [18]. A well-crafted prompt can result in more accurate, relevant, and coherent responses, effectively guiding the model to achieve the intended task [15]. Effective prompt engineering involves strategically using keywords, setting context, and providing clear instructions to engage the model’s knowledge in a specific area. This approach enhances both the utility and precision of the content produced. This process required many trials and used a considerable amount of time.

Although Custom-GPT did not stray from the context in our experience, it provided responses that varied slightly, which initially concerned us. The concern arose from observing that Custom-GPT occasionally used different phrasing or emphasized slightly different aspects of the same qualitative input across multiple runs. While its interpretations were contextually accurate, the variation raised concerns about consistency and reproducibility during early phases of testing. This prompted us to implement prompt standardization and continuous stability checks in our workflow. ChatGPT's differing responses to identical prompts can be attributed to several factors. First, its stochastic nature results from operating on probabilistic algorithms, which select words based on learned probabilities rather than fixed rules. Consequently, the same prompt can generate different responses with each interaction. Another factor is prompt sensitivity. Minor alterations in phrasing, punctuation, or context can change ChatGPT’s interpretation, leading to varied outputs. Even seemingly insignificant modifications can influence its response.

Furthermore, ChatGPT lacks true understanding or consciousness; it generates text based on learned patterns rather than genuine comprehension, which can sometimes lead to inconsistencies or inaccuracies. ChatGPT may show a degree of superficiality in its analysis, which can lead to incomplete information being extracted or a lack of sufficient analytical depth. ChatGPT faces challenges with in-depth analysis due to a lack of access to the extensive information that humans typically consider in qualitative research. This includes non-verbal cues and insights gained from experiencing the interview context and the clinical and educational environments of the participants [7]. However, this was not the case in our data, as participants provided written comments. Our observation regarding the depth of responses focused on how Custom-GPT selected quotes relevant to specific codes and themes. During our trials, Custom-GPT provided shortened versions of potential quotes submitted by participants. Upon manually reviewing and triangulating these quotes with NotebookLM, we noted that some selections would be better represented with the full quotes, as they offered more depth.

Randomness in processing also plays a significant role in results variation, as the model incorporates a degree of randomness when weighing information. By default, this randomness is set at a relatively high level, resulting in responses that may differ from previous outputs [8]. Additionally, ChatGPT’s non-deterministic nature means it does not produce fixed answers; instead, it can yield different responses to the same prompt. This variability is further enhanced by the model’s design to mimic natural language variations, making interactions feel more human-like [8]. Finally, the training data and continuous learning contribute to evolving response patterns, as the model is trained on vast amounts of data and adapts to new information over time [19].

Our experience with three different sets of qualitative data revealed that Custom-GPT largely followed the step-by-step approach of Braun & Clarke's framework. It adhered to most of the prompts and provided accurate codes and themes. However, it required continuous additional prompts to achieve the desired outcomes, particularly in elaborating on its rationale behind the decision-making process for defining the codes, themes, and quote selections.

We also found it necessary to revise the settings in Custom-GPT. While it made slight adjustments to the codes and themes, its overall understanding of the data and context remained consistent, needing only minor modifications in its recommendations. This consistency may be attributed to the relatively small volume of our data, which contained fewer words compared to the lengthy transcripts collected through interviews. We believe it is essential to keep human involvement throughout every step of the process. Moreover, triangulating the results is important. In our trials, we accomplished this by using another LLM tool, NotebookLM by Google, and we were satisfied with the assistance we received from it. One significant advantage of LLMs in qualitative analysis is their speed in processing data. A study comparing human analysts with generative AI for thematic analysis found that LLMs completed both inductive and deductive thematic analyses in about 15-25 minutes. This is approximately 96-97% less time than the human coding teams, which reported spending 492 minutes on inductive analysis and 705 minutes on deductive analysis [13].

Recommendations

Based on our experience, we recommend the following for researchers who want to use LLMs for qualitative analysis.

Before using any open-access LLM, researchers must ensure that all data files do not contain personal identifiers. This step is crucial for addressing data security concerns when working with LLMs. Due to the fact that many qualitative datasets may contain personalized information or opinion, and since these data can be utilized by open-access LLMs for training purposes in developers’ centers, it is important to inform participants in advance about how their data will be analyzed using LLMs [11]. We believe that this additional layer of transparency will enable participants to make more informed decisions about their involvement in qualitative research. Furthermore, if data security is a significant concern, researchers may prefer to download the most up-to-date model onto a local computer and use LLMs there, thereby avoiding the sharing of data with open-access LLMs’ developers [11].

Researchers should remain engaged throughout all six steps of Braun & Clarke's framework. Starting with familiarization with the data is an excellent first step before utilizing any tool for coding and theme development. This initial step enhances understanding of the qualitative data, its context, and depth before applying an LLM tool. This helps researchers maintain the accuracy of the results.

We would like to emphasize the importance of human involvement in qualitative analysis using LLMs for several key reasons. First, LLMs are effective at extracting important data points and summarizing information; however, they often struggle with conducting in-depth analysis and grasping the broader context [7]. Second, qualitative analysis entails examining the background and context of participants, especially during interviews. This includes paying attention to non-verbal cues and understanding the participants' clinical and educational environments. These aspects are often challenging for LLMs that have not directly experienced such situations to grasp [7]. Human researchers immerse themselves in data and their environment, gaining a profound understanding that LLMs can only partially provide. This deep contextual understanding is essential for fine interpretation [11]. Third, qualitative analysis typically involves using existing theories or developing new ones based on the data [13]. While LLMs can process text, they do not possess the ability to analyze and apply theoretical frameworks as thoroughly as human researchers can [7]. Furthermore, the lack of theoretical derivation is noted as a deficiency in purely ChatGPT-driven analysis [7]. Fourth, qualitative research primarily focuses on interpreting meanings and understanding human experiences, while LLMs function based on language patterns and do not possess human intuition, emotions, or lived experiences [11]. Therefore, human researchers are essential for interpreting the outputs of LLMs and contextualizing them within the research goals. The risk of automating sensemaking without substantial researcher involvement could lead to a superficial understanding of the data [11]. Fifth, LLMs are trained on extensive datasets that may include societal biases, which can be reflected or even amplified in their analyses [6]. It is essential for human researchers to identify and address these biases to ensure that the analysis is fair and accurately represents the participants and their data. Sixth, LLMs can produce nonsensical information or illogical outputs, a phenomenon referred to as "hallucinations" [6]. Human review is essential for identifying and correcting these inaccuracies, which helps ensure the reliability and validity of the analysis. Researchers are ultimately responsible for the accuracy of their findings, even when utilizing LLMs [5]. Seventh, the output of LLMs is greatly influenced by the quality of the prompts they receive [2]. To ensure that the prompts align with research questions and guide the LLMs effectively, human researchers play a crucial role in designing them [8]. Conversely, poorly constructed prompts can result in inaccurate or incomplete outputs. Eighth, qualitative research carries ethical responsibilities toward participants, particularly concerning data privacy and confidentiality [11]. Human researchers must ensure that the use of LLMs adheres to these ethical principles, especially when handling sensitive data [11]. Additionally, it is advisable to obtain ethical and legal assessments from institutional review boards before applying LLMs for qualitative data analysis. Ninth, while LLMs can provide consistency, the rigor and validity of qualitative analysis still rely on human researchers. They must make informed decisions regarding the use of LLMs, interpret their outputs, and ensure that the findings are well-supported by the data [8]. LLMs can assist with tasks such as coding and theme extraction [6]. However, human researchers are essential for reviewing the codes, guiding the thematic analysis process, and making final interpretive judgments [8].

LLMs should be viewed as tools that enhance, rather than replace, a researcher's analytical skills [8]. Although LLMs offer promising opportunities to streamline certain aspects of qualitative analysis, human involvement is crucial for ensuring the depth, contextual understanding, accuracy, ethical integrity, and overall validity of the research [8]. While LLMs can be valuable assistants, the fundamental aspects of qualitative analysis, such as interpretation, meaning-making, and critical engagement with data, require the fine skills and ethical judgment of human researchers [6]. We think that incorporating human oversight at every step and elaborating on each result and the rationale behind it will be a valuable and efficient approach.

We strongly recommend using Custom-GPT for qualitative analysis due to the limitations of general ChatGPT. Once researchers create Custom-GPT, they should pilot it with a sample of data that they have manually reviewed. This piloting phase provides an opportunity to refine instructions (prompts) within Custom-GPT, ultimately leading to better responses [7,15]. Although improvements may become evident, researchers should be aware that additional communication through extra prompts between GPT's responses may be necessary to achieve the desired outcomes [9]. We have provided our newly adjusted, simplified prompts and instructions (Table 4). These instructions and prompts were finalized during the testing phase of our data sets. They provide a step-by-step guide for extracting information from the text. Researchers may need to reuse them in the chatbox with Custom-GPT to achieve the desired outcome. Researchers can use these prompts and adapt and refine them according to their specific needs.

**Table 4:** Updated instructions and prompts for Custom-GPT in qualitative thematic analysis.

Step 1 - Define the Purpose of Your Custom-GPT
This custom GPT helps researchers follow Braun & Clarke’s 6-step process for thematic analysis. It guides users in identifying, coding, and organizing themes from qualitative texts like interviews and surveys. The tool ensures a systematic and transparent approach by promoting reflexivity and consistency.
Step 2 - Set Up Your Custom-GPT
Name: (any preferred title) Experience and Role: You are a qualitative research expert with 20 years of experience using Braun and Clarke’s six-step framework. Your role is to guide researchers through coding, categorizing, and synthesizing themes, providing clear explanations while ensuring a transparent, rigorous, and reflexive analysis.
Step 3 - Configure Braun & Clarke’s Six-Step Process
Follow the framework through each step: 1. Familiarize with the data: Start by reading and engaging deeply with the qualitative text. Summarize the dataset and identify initial patterns, emotions, and key phrases. Create a summary table Write a short paragraph (50-100 words) explaining the summary and the reasoning behind it. Please pause here and ask the user if they would like to provide additional prompts for further elaboration before you start next step. 2. Generate initial codes: Identify meaningful patterns by extracting key phrases and concepts from each section of text. Identify the three most relevant codes for each section of text. Give each code a three-word name. Write a three-line description for each code. For each code, provide original quotes provided by participants; do not modify them. Utilize the quotes in the qualitative data file exactly as they are and prefer longer quotes when available. Remove duplicate codes and quotes, then review and finalize the quote selection. Create a table with four columns: codes, three-word names, three-line descriptions, and one meaningful quote for each code. Write a short paragraph (50-100 words) explaining your reasoning for selecting these initial codes. Please pause here and ask the user if they would like to provide additional prompts for further elaboration before you start next step. 3. Identify broader themes: Look for relationships between the codes and group related codes into overarching themes. Limit the number of themes to a maximum of 10, if relevant. Create a table with two columns: codes and themes. Write a short paragraph (50-100 words) explaining your reasoning for selecting these specific themes. Please pause here and ask the user if they would like to provide additional prompts for further elaboration before you start next step. 4. Review themes for coherence: Validate that the themes are distinct, coherent, consistent, and well-supported by the data. Write a short paragraph (50-100 words) explaining your validation process and reasoning for these specific themes. Please pause here and ask the user if they would like to provide additional prompts for further elaboration before you start next step. 5. Define and name themes: Provide clear, concise names for each theme. Define each theme clearly and refine the descriptions to capture their essence, providing a detailed explanation of their meaning. For each theme, please provide selected original quotes provided by participants; do not modify them. Utilize the quotes in the qualitative data file exactly as they are and prefer longer quotes when available. Create a table with three columns: themes, description, meaning, quotes. Write a short paragraph (50-100 words) explaining your reasoning for selecting and finalizing these themes, their names, and definitions. Please pause here and ask the user if they would like to provide additional prompts for further elaboration before you start next step. 6. Generate a structured report: Present your findings with themes, supporting data, and justifications to ensure transparency and rigor. Ensure the findings align with the research questions. Please pause here and ask the user if they would like to provide additional prompts for further elaboration.
Additional Instructions and Prompts
Ask the user for clarification if the task is unclear or needs further explanation. Encourage reflexivity by prompting the user with relevant questions to critically engage with the data and justify their results. Request user feedback at each step to refine the analysis and adjust the process as needed. Adapt responses dynamically based on user input to meet specific research needs. Allow interactive user questions at any stage to foster deeper analytical thinking. Always verify your approach by referring to the attached file in the knowledge section.

Finally, adding triangulation at each step will introduce an additional layer of security to the process. The triangulation of the results from LLMs in qualitative research is essential for improving the validity, credibility, and trustworthiness of the findings when utilizing these AI tools. Triangulation involves employing multiple sources of information or different methods to verify and support research results. We used NotebookLM for triangulation instead of employing an extra human researcher. Some studies suggest using LLMs as an additional "rater" to provide confirmatory data for coding analysis [20]. The usage of LLMs for triangulation offers researchers a way to independently and efficiently verify their coding without relying solely on other human raters [8].

Limitations

There are certain limitations in using LLMs and our methodology. First, LLMs, including ChatGPT and NotebookLM, operate probabilistically, meaning that they may generate slightly different responses to the same input. This variability required additional prompting and manual intervention to refine outputs. Second, despite instructions to prompt Custom-GPT for reasoning, explainability in decision-making was lacking and again required repeated prompting and human oversight to verify the accuracy and coherence of identified codes and themes. Third, our experiences and findings are mainly relevant to qualitative research, specifically thematic analysis in medical education using text data. They may not be directly applicable to other research contexts, particularly those that require a deeply contextualized interpretation, such as ethnographic studies. Fourth, since LLMs are trained on vast datasets that may contain inherent biases, their results could reflect these biases, influencing the interpretation of qualitative data. While triangulation and human validation were conducted, subtle AI-generated biases are possible. Fifth, data privacy and security remain a concern when using open-access LLMs, as they process information externally. Even though all personal identifiers were removed from our dataset, researchers must ensure compliance with ethical guidelines and data protection policies, especially when working with sensitive information. Sixth, although LLMs significantly reduce the time required for thematic analysis, they cannot replace human judgment, especially when interpreting nuanced responses. Active human involvement remains essential throughout the analysis process. Seventh, AI technology is rapidly evolving, and newer models with improved qualitative analysis capabilities may emerge. Therefore, the methodology, instructions, and prompts outlined in this report may require ongoing updates to remain aligned with advancements in LLM capabilities. Future research should focus on refining prompt engineering techniques, developing ethical guidelines for AI-assisted qualitative research, and exploring domain-specific LLMs trained on medical education data to enhance accuracy and contextual understanding.

## Conclusions

In this report, we explored the application of LLMs in qualitative thematic analysis. Our aim in this manuscript was to contribute to the field by sharing the methodological process and tool development in a transparent and replicable manner. By developing a Custom-GPT based on Braun & Clarke’s thematic analysis framework and triangulating results using NotebookLM, we demonstrated how LLMs can assist in qualitative data interpretation while emphasizing the necessity of continuous human oversight. Our findings highlight the efficiency of LLMs in processing datasets, identifying themes, and streamlining the coding process. However, they also reveal limitations. While LLMs significantly enhance the speed and accessibility of qualitative research, they cannot replace human judgment in contextual interpretation, theoretical application, and ethical considerations. Researchers must remain actively engaged throughout all stages of AI-assisted analysis to ensure methodological rigor, validity, and accuracy. As AI technology continues to evolve, further research is needed to refine AI-assisted qualitative methodologies, develop ethical frameworks for AI-driven research, and explore domain-specific LLMs for enhanced contextual relevance in medical education. By integrating AI tools responsibly, researchers can harness their potential while maintaining the depth, accuracy, and ethical integrity that qualitative research demands.
